# Digital Radiography for Determination of Primary Tooth Length: *In Vivo* and *Ex Vivo* Studies

**DOI:** 10.1155/2015/939045

**Published:** 2015-02-23

**Authors:** Maria D. Basso, Fabiano Jeremias, Rita C. L. Cordeiro, Lourdes Santos-Pinto

**Affiliations:** ^1^Department of Pediatric Dentistry, Cascavel School of Dentistry, Universidade Estadual do Oeste do Paraná (UNIOESTE), Rua Universitária 2069, 85819-110 Cascavel, PR, Brazil; ^2^Department of Pediatric Dentistry and Orthodontics, Araraquara School of Dentistry, Universidade Estadual Paulista (UNESP), Rua Humaitá 1680, 14801-903 Araraquara, SP, Brazil

## Abstract

*Background.* Methods for determining the root canal length of the primary tooth should yield accurate and reproducible results. *In vitro* studies show some limitations, which do not allow their findings to be directly transferred to a clinical situation. *Aim.* To compare the accuracy of radiographic tooth length obtained from *in vivo* digital radiograph with that obtained from *ex vivo* digital radiograph. *Method.* Direct digital radiographs of 20 upper primary incisors were performed in teeth (2/3 radicular resorption) that were radiographed by an intraoral sensor, according to the long-cone technique. Teeth were extracted, measured, and mounted in a resin block, and then radiographic template was used to standardise the sensor-target distance (30 cm). The apparent tooth length (APTL) was obtained from the computer screen by means of an electronic ruler accompanying the digital radiography software (CDR 2.0), whereas the actual tooth length (ACTL) was obtained by means of a digital calliper following extraction. Data were compared to the ACTL by variance analysis and Pearson's correlation test. *Results.* The values for APTL obtained from *in vivo* radiography were slightly underestimated, whereas those values obtained from *ex vivo* were slightly overestimated. No significance was observed (*P* ≤ 0.48) between APTL and ACTL. *Conclusion.* The length of primary teeth estimated by *in vivo* and *ex vivo* comparisons using digital radiography was found to be similar to the actual tooth length.

## 1. Introduction

The anatomy knowledge, degree of root curvature and relationship between tooth and surrounding structures, is essential to endodontic working length determination. Errors in the determination of the tooth length may cause endodontic instruments to go beyond the apical foramen, resulting consequently in extravasation of irrigating solutions and restorative material into the periradicular tissues. In primary teeth, root canal instrumentation is risky as it can damage the permanent tooth germ.

Therefore, the first periapical radiography for diagnosis is highly relevant as it allows determining the apparent tooth length. This measurement is important to determine the working length, which is crucial for a successful treatment due to the need for complete disinfection without periapical tissues harming [1]. However, it is often difficult to obtain a reliable diagnostic procedure in children because of their poor cooperation and limited access to the mouth [2].

However, it is not always possible to determine accurately the radiographic tooth length because of anatomical variations in the apical foramen, which is not visualised in the radiograph. In addition, roots often present different degrees of curvature or anatomical structures superposition. Presence of periapical pathology, degree of pathological or physiological resorption, and presence of permanent successor tooth are also factors complicating the working length determination of primary teeth [3]. Clinically, the radiographic apex is used as reference point [4]. However, because apex does not always coincide with the actual apical foramen position, there is a difference between apparent tooth length (APTL) and actual tooth lengths (ACTL) in the majority of the cases [4].

Although the accuracy in the endodontic length determination using digital radiography had been found to be inferior to that of conventional radiography [5–8], some studies demonstrate that the former is equal or even superior to the latter for obtaining length measurements [2, 9–11]. The majority of such studies were carried out* in vitro*, but scattered radiation effects, osseous trabeculae superposition, and difference in bone density are all factors present in the patient's radiograph, which do not allow* in vitro* findings to be directly transferred to a clinical situation [6]. Therefore, the aim of this study was to compare the accuracy of radiographic tooth length obtained from *in vivo* digital radiograph with that obtained from *ex vivo *digital radiograph.

## 2. Material and Methods

### 2.1. Sample Selection and Preparation

The radiographs have been obtained by one calibrated operator using radiographic positioner and sensor number 1 (size of outer sensor: 37 × 24 mm; size of active area: 30 × 20 mm; size of thickness: less than 5 mm) of the Computed Dental Radiography System (CDR, Schick Technologies Inc., Long Island City, NY, USA) according to paralleling technique. The X-ray equipment (Gnatus XR 6010, Ribeirão Preto, SP, Brazil) operating at 60 kvp and 7 mA also was used. The 0.3-second radiation time and the 30 cm distance between target and sensor were determined by a pilot study following critical analysis of the image quality.

After approval by the local research ethics committee (protocol number 18/98), twenty (20) upper central and lateral primary teeth were selected from those patients who attended the Clinic Pediatric Emergency Service for extraction. We selected incisors, from 3-to-5-year-old children, that presented 2/3 of physiological radicular resorption. Behavior and cooperation were a problem for few of the children, but we had the parents support because they have understood the need for the treatment to be performed. After obtaining the radiographs, the teeth were extracted, cleaned, and stored in 4% formaldehyde solution.

**Figure 1:**
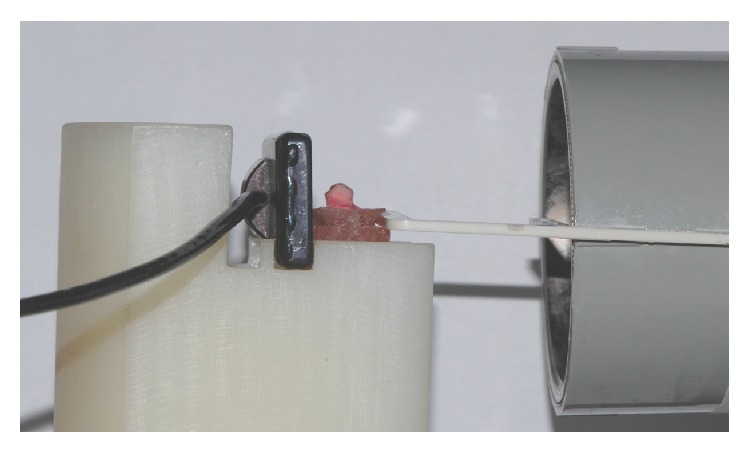
A template was used to simulate the radiographic technique of paralleling.* Ex vivo* study.

With regard to the* ex vivo* study, teeth were individually mounted in acrylic resin blocks by using a template. These blocks were adapted to a radiographic positioner specially made to ensure standardisation of both angulation and distance between radiation source, object, and sensor. A groove made in the resin block allowed adapting a guiding pole for positioning of the X-ray cylinder, thus enabling simulation of the radiographic technique of paralleling (Figure 1).

### 2.2. Examination Methods

The digital images were recorded in TIFF format and stored in a USB memory stick, with the apparent tooth length (APTL) measurements being directly performed on the high-resolution colour screen with 100% magnification. The measurement system used was the electronic ruler accompanying the digital radiography software (CDR, version 2.0, Schick Technologies, Inc., Long Island City, NY, USA) in which only two clicks are enough: the first at the visible extremity of the crown and the second at the root apex. Prior to the measurements, the electronic ruler was calibrated by measuring the radiographic image of a known size object (Kerr-K File number 30, Le Fils d'Auguste Maillefer SA, Switzerland). Enhancement features such as contrast and brightness were not adjusted during the on-screen measurements. Measurements of radiographic images were performed twice at a 2-week interval by two calibrated examiners: a Paediatric Dentistry Professor with experience in using the digital radiography system (Observer A) and a postgraduate student with limited experience (Observer B).

Two measurements corresponding to the distance between crown extremity and anatomical root apex of every extracted tooth were performed by using a digital calliper (Mitutoyo Corporation, Japan) and the results were recorded as being the actual tooth length (ACTL).

### 2.3. Statistical Analysis

The comparison of the tooth length measurements resulting from* in vivo* and* ex vivo* digital images with actual tooth lengths was carried out by means of variance analysis at significance level of 0.05. Pearson's correlation test (*r*) was used to evaluate the correlation between the first and second measurements performed by both examiners.

## 3. Results

The results shown in Table 1 have demonstrated that the values of apparent tooth length in radiographs obtained* in vivo* were slightly underestimated, whereas those obtained in* ex vivo* conditions were slightly overestimated. Variance analysis demonstrated that there was no statistically significant difference (*P* ≤ 0.48) between actual tooth length (ACTL) and apparent tooth length (APTL) obtained from radiographs of patients and extracted teeth mounted in resin blocks.

The results regarding Pearson's correlation coefficient (*r*) have shown excellent intra- and interexaminer correlations as well as a good correlation between actual tooth length and radiographic tooth length in the* in vivo* study. The same correlations were all excellent in the* ex vivo* study. However, the correlation between the results obtained in both studies was found to be moderate only (Table 2).

The tooth length was accurate in 100% and 65% of the cases for tolerance of ±1.0 mm in* ex vivo* and* in vivo* studies, respectively. When tolerance was ±0.5, these figures, however, decreased to 65% and 30% in* ex vivo* and* in vivo* radiographies, respectively (Table 3).

## 4. Discussion 

Methods for determining the root canal length of the primary tooth should yield accurate and reproducible results [12]. In primary teeth, technical failures can induce overinstrumentation, overfilling, and root perforation/fracture or even damage the permanent tooth [2]. On the other hand, underfilling is also a risk factor accounting for ongoing disease [13, 14].

In order to avoid repeated exposures to radiation, experimental models with extracted teeth were used in studies on the accuracy of measurements from radiographic images. However, the absence of images of periodontal ligament and osseous trabeculae and the presence of superposition of anatomical structures in the* in vitro* studies are factors making it difficult to transfer the results into clinical situations [4, 7].

Distortions and amplifications of digital images can occur due to geometrical variations, such as angulation, distance between sensor and tooth, and distance between X-ray source and tooth. These variations can be minimised by obtaining a radiographic image of a radio-opaque object of known size so that its actual dimensions can be used as a benchmark for calibration of the file directly on the computer screen [15]. In the present study, the image of an endodontic file was used for such calibration by placing it in parallel with the tooth to be measured in order to confirm the calibration accuracy [15].

The most common method used for working length determination in endodontics involves the observation of root length by means of preoperative radiography and evaluation of a radiograph of a file inside the root canal, always adopting estimated lengths of 1 mm and 2 mm beyond the radiographic apex for permanent teeth [9, 16, 17] and primary teeth [17], respectively.

The difference between actual and radiographic tooth lengths was termed as estimation error. Measurements obtained with variation up to ±1 mm were regarded as accurate because the practitioner in general will introduce a file of 1 mm less than the radiographic tooth length. With estimation errors within these values, the files would not exceed the root apex.

The intraexaminer agreement was excellent, similar to studies evaluating maxillary permanent root lengths in cadaver sections [18], curved canal lengths of extracted permanent teeth [19], and primary tooth length [20]. A high interexaminer agreement was also observed in the present study, which was demonstrated through digital radiographic techniques by identifying the endodontic file tips inside the root canal length in both* in vivo* and* in vitro* conditions [6, 7]. However, the correlation between* in vivo* and* ex vivo *results was found to be moderate only.

Despite the high correlation between the* in vivo* digital radiographic measurements and the actual tooth length, this radiographic method indeed underestimated the tooth length (Table 1). A possible explanation is that the primary tooth presented physiological resorption in most radiographic images (81%), and an underlying permanent tooth germ caused image superimposition. Consequently, the root apex was not clearly identified. Underestimation of both permanent tooth canal and file length in digital radiographic examinations was reported [10, 21]. Likewise, overestimation was noted in the* ex vivo *study. High values for the actual canal length were also demonstrated in radiographic determination of the curved canal length [11, 19].

In the present study, the results showed that 65% of the measurements were accurate as in the* in vivo* determination of the tooth length. However, when a 0.5 mm tolerance was considered satisfactory, only 30% of the measurements were accurate (Table 3). Ong and Ford [9] reported 63% accuracy with such a variation. In* ex vivo* conditions, 65% of the radiographs were found to be accurate for 0.5 mm tolerance, whereas 100% accuracy was found for 1 mm tolerance (Table 3). Similar values were reported by Ong and Ford [9] that found 70% of accuracies with 0.5 mm. Studies with cadavers also reported similar results, with mean estimation error of 0.70 and measurements ranging from 1.3 (underestimation) to 1.5 (overestimation) [5], which were close to the values found by* in vivo* studies.

Diagnostic methods requiring children's cooperation are less reliable compared to those involving adult patients. Determination of the working length can be achieved by means of electronic resources, such as an electronic apex locator [3, 11, 22, 23]. However, one should consider that children with endodontic problems usually had previous pain experience, which may result in behaviour problems. Therefore, the treatment has to be performed as quickly as possible for diagnosis and therapeutic decision making. Digital radiography is a fast technique allowing paralleling and reduction in radiation dose* per* image, besides being comfortable to the patient and yielding data which can be quickly analysed by software [15, 24].

Our findings suggested that the tooth length obtained with straight-line measurement on direct digital radiographs was similar to the actual tooth length. An electronic ruler was the best measurement instrument in an endodontic length study developed by Vandre et al. [25] and no significant difference was found between canal length estimates using multiple measurements points or a 2-click measurement technique [19, 25]. Thus, the current results support the clinical recommendation that digital radiographs should be taken to determine root length.

## 5. Conclusion 

This study reveals that the length of primary teeth estimated by* in vivo* and* ex vivo* studies using digital radiographs was found to be similar to the actual tooth length. However it is possible to determine primary tooth length in digital radiography using on-screen measurements with a reasonable degree of accuracy.

In brief we have the following:

- it is possible to determine primary tooth length in digital radiography using on-screen measurements with a reasonable degree of accuracy;
- digital radiography exposes the child to less radiation than a standard film radiograph and reduces the working time, especially in post-trauma and difficulty of management;
- digital radiography should be taken to determine root length reducing periapical injury and possible damage to the permanent successor.

## Figures and Tables

**Table 1:** Means, standard deviations (SD), and minimum and maximum values of tooth length. Differences between radiographic tooth length and actual tooth length (ACTL).

	Mean (mm)	Std. deviation	Minimum (mm)	Maximum (mm)
*In vivo *	10.66	1.61	7.98	13.25
*Ex vivo *	11.24	1.50	8.45	13.43
ACTL	11.00	1.41	8.00	13.40
*In vivo* mean difference	0.34	1.17	−1.27	2.70
*Ex vivo* mean difference	−0.24	0.40	−0.80	0.75

**Table 2:** Interexaminer agreement and intraexaminer agreement and tooth length accuracy concordance, Pearson's correlation values.

X-ray	Examiner	Measurement session	Mean error of measurement (mm)	*P* value	Pearson's correlation (*r*)			
					Intraexaminer	Interexaminer	APTL × ACTL	*In vivo *×* ex vivo *
*In vivo *	A	1	0.39	0.44	0.92	0.89	0.71	0.68
		2	0.51					
	B	1	0.09	0.10	0.91			
		2	0.37					
*Ex vivo *	A	1	−0.12	0.55	0.95	0.98	0.96	
		2	−0.07					
	B	1	−0.38	0.88	0.96			
		2	−0.37					

**Table 3:** Percentage of accuracy in the tooth length determination.

	APTL-ACTL			
	*In vivo *		*Ex vivo *	
	Mean variation ≤ 0.5 mm	Mean variation ≤ 1.0 mm	Mean variation ≤ 0.5 mm	Mean variation ≤ 1.0 mm
% equivalence	30	65	65	100
% overestimation	30	10	30	—
% underestimation	40	25	5	—
